# Hypercholesterolemia Enhances T Cell Receptor Signaling and Increases the Regulatory T Cell Population

**DOI:** 10.1038/s41598-017-15546-8

**Published:** 2017-11-15

**Authors:** Reiner K. W. Mailer, Anton Gisterå, Konstantinos A. Polyzos, Daniel F. J. Ketelhuth, Göran K. Hansson

**Affiliations:** Cardiovascular Medicine Unit, Department of Medicine, Karolinska Institutet, Stockholm, Sweden

## Abstract

Hypercholesterolemia promotes the inflammation against lipoproteins in atherosclerosis. Development of atherosclerosis is affected by the balance between pro-inflammatory effector T cells and anti-inflammatory regulatory T (Treg) cells. However, phenotype and function of T cell subpopulations in hypercholesterolemia remain to be investigated. Here, we found that cholesterol-containing diet increased the expression of the Treg cell lineage-defining transcription factor FoxP3 among thymocytes and splenocytes. Hypercholesterolemia elevated the FoxP3 expression level and population size of peripheral Treg cells, but did not prevent enhanced proliferation of stimulated T cells. Moreover, cholesterol supplementation in diet as well as in cell culture medium promoted T cell antigen receptor (TCR) signaling in CD4+ T cells. Our results demonstrate that hypercholesterolemia enhances TCR stimulation, Treg cell development as well as T cell proliferation. Thus, our findings may help to understand why hypercholesterolemia correlates with altered CD4+ T cell responses.

## Introduction

Cardiovascular disease (CVD) is the leading cause of deaths worldwide. CVD is the result of a chronic inflammation of the arterial wall, where the accumulation of lipoprotein particles elicits the activation of innate and adaptive immune cells. In search for therapeutic mechanisms to prevent CVD development, many studies have focused on regulatory T (Treg) cells that inhibit immune responses in multiple cell types, such as macrophages, antigen presenting cells (APCs) and T cells^1^. This immunosuppressive effect mediated by Treg cells reduces experimental atherosclerosis^2,3^. However, experimental atherosclerosis is paradoxically associated with increasing Treg cell populations^4^. While the reason for this increase remains elusive, its failure to prevent disease development has been attributed to impaired cell adhesion, differentiation and plasticity^4–6^.

In general, T cells scan for antigens through serial and transient interaction with surrounding APCs. During this, their TCRs and co-receptors are redirected via capping, an antigen-independent process where pre-formed lipid rafts or nanoclusters are re-organized^7^. Lipid raft integrity is crucial for efficient T cell activation^8–10^. Cholesterol is known to stabilize these membrane domains and binds to the TCRβ-chain to facilitate TCR dimerization; thus increasing avidity towards antigen^11^. In contrast, derivatives of cholesterol that prevent TCR multimerization or disrupt membrane organization are reported to inhibit TCR signaling, to limit antigen-specific responses and to influence T cell differentiation^12–14^. However, some studies reported that cholesterol deprivation enhances TCR signaling^15–17^, suggesting that cholesterol-mediated effects are strongly influenced by the experimental setup.

Initiation and termination of TCR signaling are mediated through differential formation, mobility and internalization of lipid rafts^18^. Following TCR stimulation, various endocytic mechanisms decrease the surface expression of the CD3 complex on the plasma membrane^19,20^. Beside the effect of cholesterol on plasma membrane dynamics, cholesterol metabolism also supports the proliferation of activated T cells as well as the size and function of the Treg cell population^21–23^. Moreover, homeostatic TCR signaling allows Treg cells to maintain their dynamic proliferative character and to express high levels of their lineage-defining transcription factor FoxP3^24,25^. Despite the link between hypercholesterolemia and TCR stimulation and the importance of homeostatic TCR stimulation for Treg cells, the ability of hypercholesterolemia to affect FoxP3 expression and the Treg cell population has not been investigated so far.

In this study, we demonstrate that hypercholesterolemia increased the homeostatic TCR signaling in CD4+ T cells. By this, hypercholesterolemia increased the development of FoxP3+ T cells in the thymus and elevated the FoxP3+ Treg cell population in the periphery. In parallel, hypercholesterolemia led to enhanced CD3 internalization and proliferation of stimulated T cells. Moreover, cholesterol supplementation in diet as well as in cell culture medium increased the TCR signaling strength in naïve CD4+ T cells.

## Materials and Methods

### Animals

Experiments have been carried out on in-house bred C57BL/6 J mice, *Ldlr*−/− mice (B6.129S7-*Ldlr* ^*tm1Her*^/J, The Jackson Laboratory), Nur77^GFP^ mice (C57BL/6-Tg(Nr4a1-EGFP/cre, The Jackson Laboratory) or hemizygous DEREG FoxP3 reporter^3^, kindly provided by Dr. T. Sparwasser (Institute for Infection Immunology, TWINCORE, Hannover, Germany); an overview of genetic modifications of used mouse strains is provided in (Supplemental Table 1). All experimental animals were bred under standard housing conditions, were between 8–10 weeks of age, were genotyped and were randomly selected into treatment groups. Mice were fed cholesterol-free standard chow diet (SCD) (R70, Lantmännen, Sweden), 0.15% cholesterol containing Western diet (WD) (R638, Lantmännen, Sweden) or 1.125% cholesterol containing WD (D12108C, Research Diets, USA) for 4, 12 or 24 weeks; detailed list of ingredients is provided in (Supplemental Table 2).

### Cell culture

T cells derived from spleens of wild type, *Ldlr*−/− and Nur77^GFP^ mice were isolated with Naïve CD4+ T cell Isolation Kit (Miltenyi) and cultured in serumfree X-VIVO 15 Medium (Lonza). For *in vitro* stimulation experiments cells were incubated with 1 µg/ml soluble anti-CD3 antibody and 0.5 µg/ml soluble anti-CD28 antibody for 1–2 days, if not stated otherwise in the figure legends. In experiments using solubilized cholesterol supplementation, cholesterol (Sigma) was pre-dissolved in acetone and used at a final concentration of 9 µg/ml to avoid unspecific and/or cytotoxic effects of cyclodextrin treatment^26^.

### Proliferation assay

Splenocytes derived from SCD or WD fed mice were stimulated *in vitro* with variable plate-bound anti-CD3 antibody concentrations and soluble anti-CD28 antibody (1 µg/ml) for two days followed by a 12 h pulse with 1 µCi ^3^H-thymidine per well. Cells were harvested (Tomtec) and thymidine uptake was assessed in a beta counter (PerkinElmer).

### Suppression assay

Splenocytes derived from mice fed SCD or WD for 4 weeks were used to isolate suppressor T cells, untouched responder T cells and APCs (CD4- fraction) using CD4+ CD25+ Regulatory T cell Isolation Kit (Miltenyi). Suppression assay was performed as reported before^27^. Briefly, carboxyfluorescein succinimidyl ester (CFSE) labeled responder cells proliferated in the presence of irradiated APCs (30 Gy), soluble anti-CD3 antibody (1 µg/ml) and anti-CD28 antibody (0.5 µg/ml) and variable amounts of suppressor T cells. CFSE dilution was measured after 4 days with three technical replicates and suppression was calculated by subtracting percentage of proliferating cells in suppression from percentage of proliferating cells alone; highest suppression value was set to 100%.

### Flow cytometry

FACS analysis of primary T cells obtained from murine lymphoid organs was performed on cells within the lymphocyte gate of forward/side scatter plots, excluding doublets and dead cells (LIVE/DEAD Fixable Aqua Dead Cell Stain Kit, Invitrogen) and gated on CD4+ T cells. Fc receptor binding was prevented by anti-CD16/CD32 blockade (clone: 2.4G2, BD Biosciences) and unspecific binding was excluded by isotype control stainings (BD Biosciences). Intracellular staining was performed using the FoxP3 staining kit (eBioscience). Following fluorescent primary antibodies were purchased from BD Biosciences: PE-Cy7 labeled anti-CD25 (PC61), PE-Cy5 labeled anti-CD8α (53-6.7), APC-H7 labeled anti-CD4 (GK1.5), PE/PerCP/Pacific Blue labeled anti-CD3ε (500A2); purchased from BioLegend: BV421 labeled anti-Ki-67 (16A8) or purchased from eBioscience: APC labeled anti-Nrp-1 (3DS304M), PE/Pacific Blue/APC labeled anti-FoxP3 (FJK-16s, NRRF-30), PerCP-eFluor710 labeled anti-Helios (22F6). Events were acquired on CyAn ADP Analyzer (Beckman Coulter) and data were analyzed with FlowJo v10.0.7 software (Treestar).

### Immunoblotting

Western blots of FoxP3 were performed as described before^27^; intensity of FoxP3 bands detected with anti-FoxP3 antibody (eBio7979, eBioscience) was analyzed using ImageJ 1.48 v (NIH) software and values are calculated relative to the corresponding intensity of heat shock protein 70 (HSP70) bands detected with anti-HSP70 antibody (1B5, Assay designs).

### Statistics

Values are expressed as mean ± SEM unless otherwise stated. Statistical analysis of data was performed, as indicated in the figure legends. Data with normal distribution were analyzed with two-tailed Student’s t-test and one-way ANOVA followed by Bonferroni’s multiple comparison *post hoc* test for two and more groups, respectively. Data, where normal distribution could not be assumed, were analyzed with two-tailed Mann–Whitney *U* test and Kruskal-Wallis ANOVA followed by Dunn’s multiple comparison *post hoc* test for two and more groups, respectively. Paired data that were normally distributed were analyzed with paired two-tailed Student’s t-test and repeated measures ANOVA for comparison of two and more groups, respectively. Paired data, where normal distribution could not be assumed, were analyzed with two-tailed Wilcoxon matched-pairs test. For comparisons of more than two groups with two variables two-way ANOVA followed by Bonferroni’s multiple comparison *post hoc* test was performed. Correlations were calculated using Spearman’s rank test. Differences were considered significant at *P*-values < 0.05. All statistical analyses were performed using GraphPad Prism v5.03 software.

### Study approval

This study was carried out in accordance with the recommendations of Directive 2010/63/EU of the European Parliament on the protection of animals used for scientific purposes. The protocol was approved by the Stockholm Norra regional ethical board.

## Results

### Dietary cholesterol intake increases FoxP3 in secondary lymphoid organs

**Figure 1:**
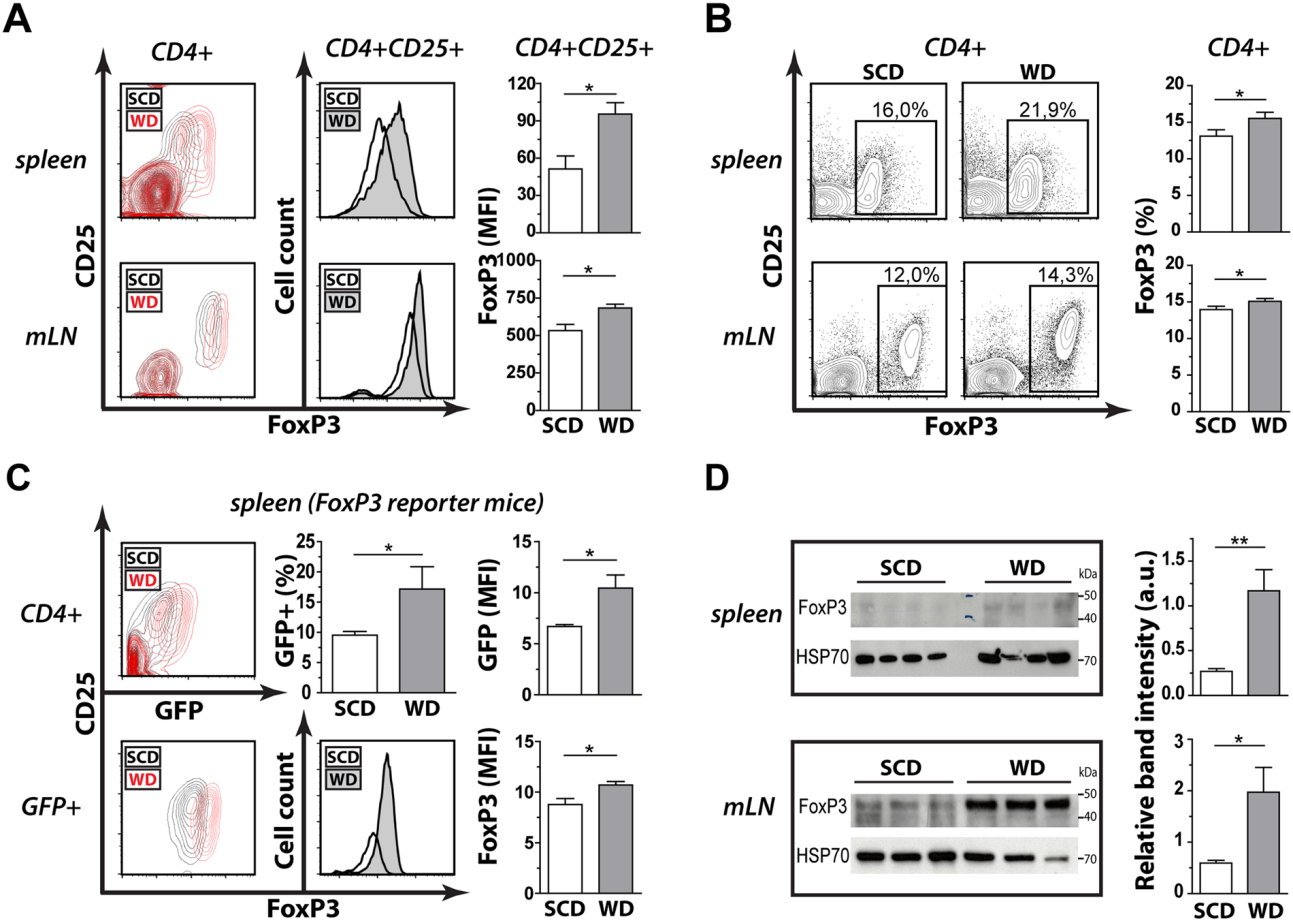
Dietary-induced hypercholesterolemia increases cellular FoxP3 expression levels and Treg cell populations in spleen and mesenteric lymph nodes. FoxP3 expression in mice fed cholesterol-free standard chow diet (SCD) or 0.15% cholesterol-containing Western diet (WD) for 4 weeks. (**A**) Representative contour plots of live CD4+ T cells (SCD black lines; WD red lines) as well as histograms (SCD in white; WD in gray) and bar graphs of live CD4+ CD25+ T cells from spleen (upper panel) and mesenteric lymph nodes (mLN) (lower panel) derived from SCD (n = 4) or WD (n = 5) fed mice. (**B**) FoxP3+ Treg cell populations among live CD4+ T cells as in (**A**). Data for spleen (n = 21) and mLN (n = 10) from six and four independent experiments are shown, respectively. (**C**) Representative contour plots (SCD black lines; WD red lines) as well as histograms and bar graphs (SCD in white; WD in gray) of FoxP3 reporter mice (DEREG) gated on live CD4+ T cells (upper panel) or live CD4+ GFP+ Treg cells (lower panel) derived from spleens of SCD (n = 5) or WD (n = 3) fed mice. One representative experiment out of three is shown. (**D**) FoxP3 immunoblots among splenocytes (upper panel) and mesenteric lymph nodes (mLN) (lower panel) and quantification of relative band intensities of SCD or WD fed mice. All data are expressed as mean ± SEM; two-tailed Mann Whitney U test was performed for statistical analysis; *p < 0.05; **p < 0.01.

To investigate the effect of cholesterol-containing Western diet (WD) on the T cell compartment in wild type mice, we analyzed the population size of different subsets in spleen and mesenteric lymph nodes. We found that four weeks of WD elevated the expression level of FoxP3 among CD4+ CD25+ T cells and increased the FoxP3+ Treg cell population size in both organs compared to standard chow diet (SCD) fed littermates (Fig. 1A,B). In contrast, no significant changes were detected for body weight, weight and cellularity of lymphoid organs (Supplemental Table 3) and total CD4+ or CD8+ T cell populations (Supplemental Fig. 1). These results were confirmed with different antibody clones against FoxP3 (data not shown) as well as FoxP3 reporter mice (Fig. 1C). Furthermore, FoxP3 immunoblots revealed that WD increased the FoxP3 expression among total splenocytes and mesenteric lymph node cells with comparable apparent molecular weight (Fig. 1D, Supplemental Fig. 2), suggesting that WD fosters the expression of functional FoxP3 in secondary lymphoid organs.

### Increasing hypercholesterolemia correlates with Treg cell population size in spleen

**Figure 2:**
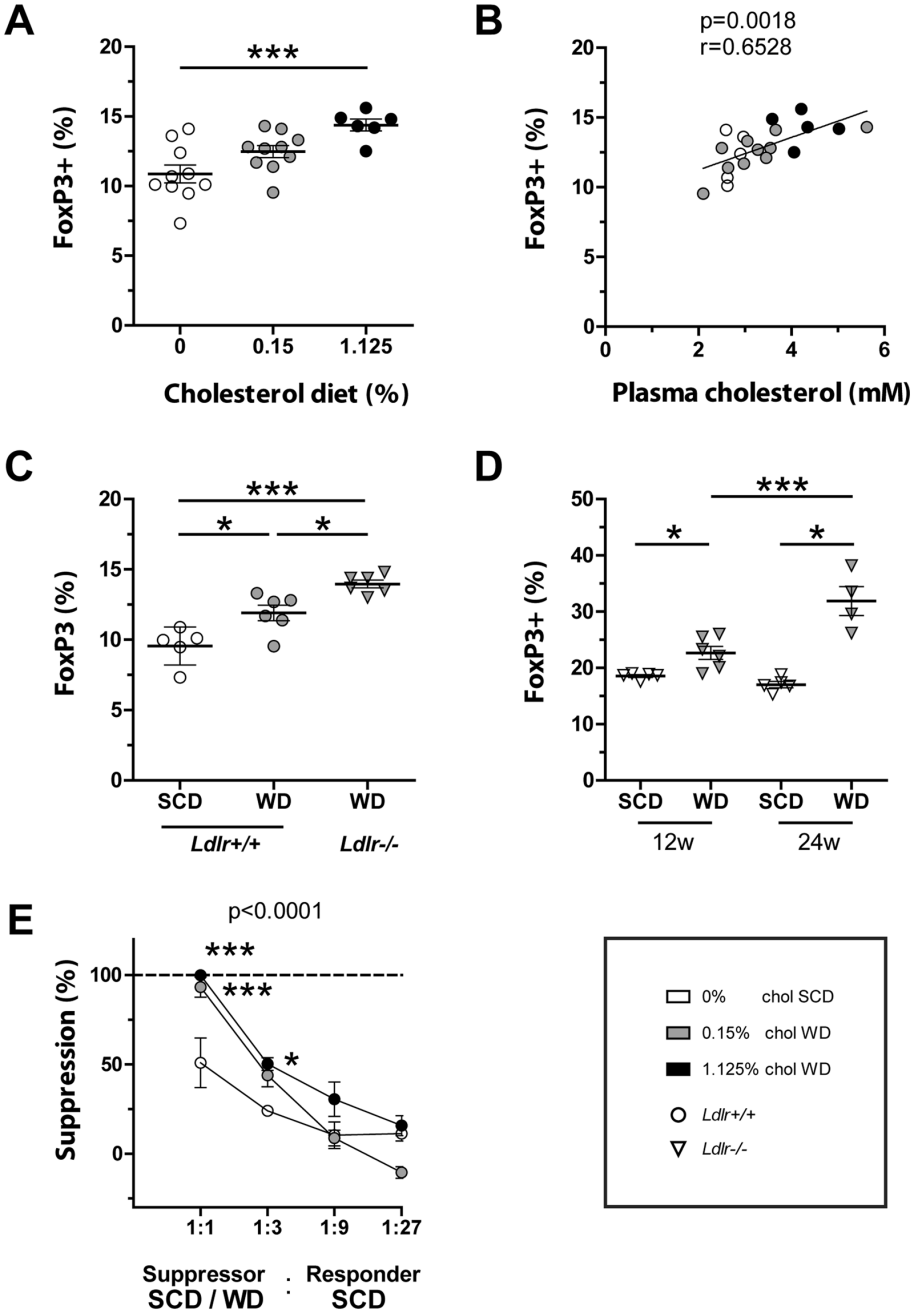
The population of functional Treg cells correlates with hypercholesterolemia. (**A**) Splenic FoxP3+ Treg cell populations among live CD4+ T cells following 4 weeks of cholesterol-free (white) standard chow diet (SCD), 0.15% (gray) or 1.125% (black) cholesterol-containing Western diet (WD) in wild type mice. One-way ANOVA with Bonferroni’s multiple comparison *post hoc* test was performed for statistical analysis. (**B**) Splenic FoxP3+ Treg cell populations as in (**A**) against plasma cholesterol concentrations; r = Spearman’s rank correlation coefficient. (**C**) Splenic FoxP3+ Treg cell populations among live CD4+ T cells in wild type (*Ldlr* +/+, circles) and LDL receptor knockout (*Ldlr*−/−, triangles) mice fed SCD (white) or 0.15% cholesterol-containing WD (gray) for 4 weeks. One-way ANOVA with Bonferroni’s multiple comparison *post hoc* test was performed for statistical analysis. (**D**) Splenic FoxP3+ Treg cell populations among live CD4+ T cells in *Ldlr*−/− mice fed SCD or WD for 12 and 24 weeks, respectively. One-way ANOVA with Bonferroni’s multiple comparison *post hoc* test was performed for statistical analysis. (**E**) Suppression of CD4+ responder T cell proliferation by CD4+ CD25+ suppressor T cells isolated from mice treated as in (**A**); CFSE-labeled responder T cells and irradiated APCs are derived from SCD fed mice; maximum suppression was set to 100%. Representative data from one out of two experiments with three technical replicates per condition are shown. Two-way ANOVA with Bonferroni’s multiple comparison *post hoc* test was performed for statistical analysis, displayed p-value indicates the significant effect of the diet on the suppressive capacity. (The p-value for the effect of the dilution of suppressor cells is not shown). All values are expressed as mean ± SEM; color and shape of data points indicate treatment and genotype of mice, as described in the legend; *p < 0.05; ***p < 0.001.

Next, we tested whether increasing the cholesterol content of the diet further expanded the Treg cell population. FoxP3 expression among CD4+ T cells gradually increased with dietary cholesterol and correlated linearly with plasma cholesterol concentrations (Fig. 2A,B, Supplemental Fig. 3A). The increase of FoxP3 expression in CD4+ T cell was further elevated in WD fed mice that developed more severe hypercholesterolemia due to low-density lipoprotein receptor (LDLr) deficiency (*Ldlr*−/−) (Fig. 2C, Supplemental Fig. 3B), which is in line with previous reports^4^. Dietary cholesterol, treatment period, and LDLr deficiency had an additive effect and resulted in unusually excessive Treg cell populations that comprised one third of the CD4+ T cell compartment (Fig. 2D), but had no effect on Th1 or Th17 differentiation in the spleen^28^.

### Hypercholesterolemia increases functional thymic-derived Treg cells

**Figure 3:**
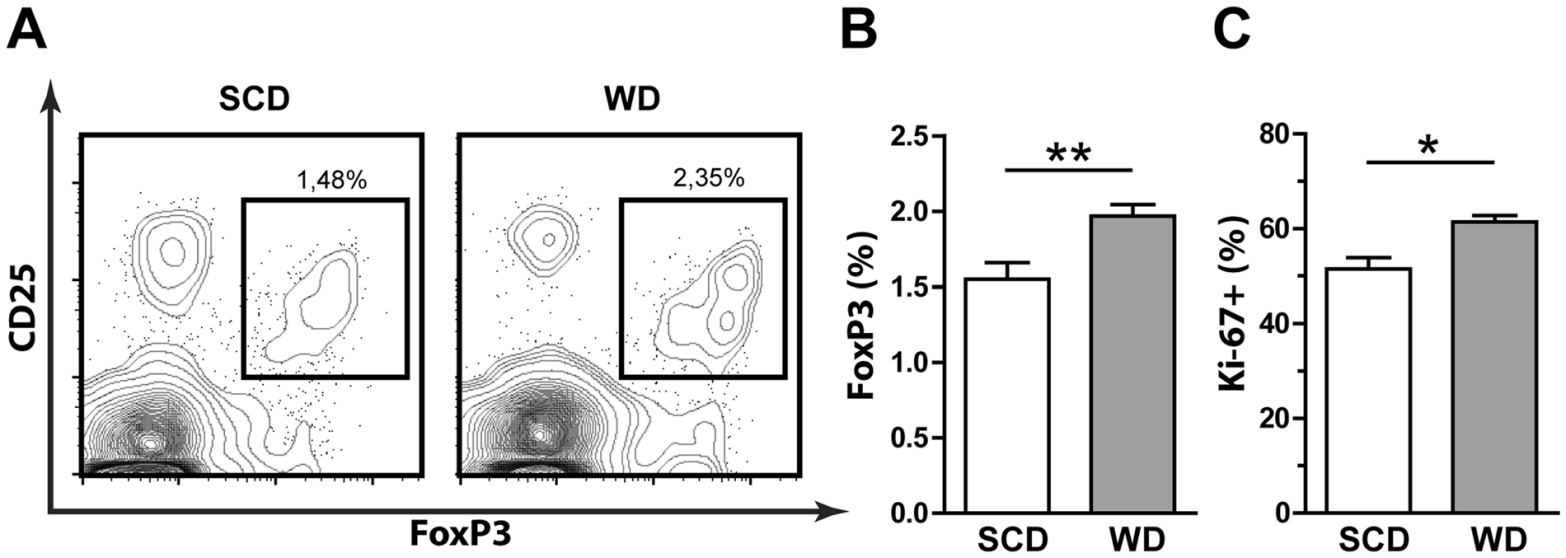
Hypercholesterolemia increases population size and proliferation of FoxP3+ T cells in the thymus. Flow cytometry analysis of the thymus following 4 weeks of cholesterol-free standard chow diet (SCD) or 0.15% cholesterol-containing Western diet (WD). (**A**) Representative contour plots of live CD4+ thymocytes from SCD or WD treated mice and percentages of the CD4+ CD25+ FoxP3+ population are shown. Percentage of (**B**) CD4+ CD25+ FoxP3+ among CD4+ thymocytes in SCD (white, n = 6) or WD (gray, n = 7) fed mice and (**C**) Ki-67+ among CD4+ CD25+ FoxP3+ thymocytes in SCD (white, n = 3) or WD (gray, n = 4) fed mice. Values are expressed as mean ± SEM; ; two-tailed Mann Whitney U test was performed for statistical analysis; *p < 0.05, **p < 0.01.

In line with elevated FoxP3 expression, CD4+ CD25+ Treg cells obtained from mice fed cholesterol-containing diets showed an increased capacity to suppress the proliferation of normocholesterolemic CD4+ CD25− responder cells (Fig. 2E). To clarify whether peripheral differentiation of TGFβ-dependent inducible Treg (iTreg) cells was the cause of hypercholesterolemia-mediated increase of Treg cell populations, we analyzed markers that were reported to distinguish thymic-derived Treg (tTreg) cells from iTreg cells. However, following treatment with variable dietary cholesterol content no significant changes were observed in the expression levels of Helios or Neuropilin-1 (Nrp-1) in Treg cells (Supplemental Fig. 4A,B). Moreover, hypercholesterolemia did not affect the proliferation of splenic Treg cells and the addition of serum derived from WD fed mice did not promote iTreg cell generation *in vitro* (Supplemental Fig. 4C and data not shown). In contrast, WD increased the size and proliferation of the FoxP3+ population among thymocytes (Fig. 3A–C), whereas no significant change in viability or population size of single positive and double negative thymocytes was observed (Supplemental Fig. 5A). Notably, increased FoxP3 expression levels among thymocytes did not reach statistical significance, suggesting that FoxP3 expression is stabilized and promoted after thymic egress (Supplemental Fig. 5B).

### Net support of T cell proliferation in Western diet fed mice

**Figure 4:**
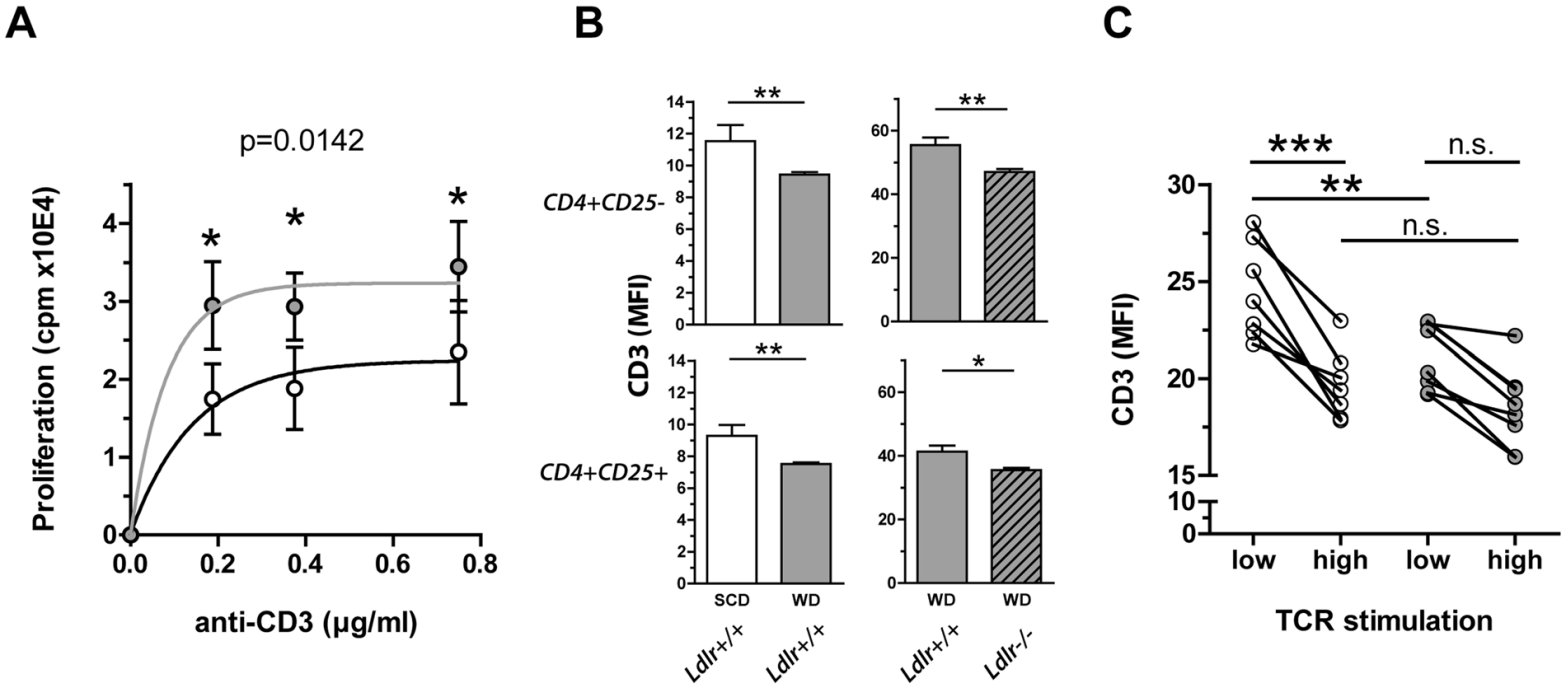
Enhanced proliferation and TCR internalization of CD4+ T cells in hypercholesterolemia. (**A**) Proliferation of splenocytes derived from 4 weeks cholesterol-free standard chow diet (SCD, white) or 0.15% cholesterol-containing Western diet (WD, gray) fed mice stimulated with different anti-CD3 concentrations; pooled data from three independent experiments with four technical replicates for each data point are shown (n = 9). Values are expressed as mean ± SEM; two-way ANOVA with Bonferroni’s multiple comparison *post hoc* test was performed for statistical analysis; displayed p-value indicates the significant effect of the diet on T cell proliferation. (The p-value for the effect of the antibody dilution is not shown). (**B**) CD3 internalization in splenic CD4+ CD25− (upper panel) or CD4+ CD25+ (lower panel) T cells derived from wild type mice (*Ldlr*+/+) fed SCD (white) or WD (gray) for 4 weeks, assessed by anti-CD3 surface stainings utilizing Pacific Blue-conjugated antibody (n = 8) (left panel) or derived from *Ldlr*+/+ (plain) and LDL receptor knockout (*Ldlr*−/−) mice (hatched) fed WD for 4 weeks, assessed by anti-CD3 surface stainings utilizing phycoerythrin-conjugated antibody (n = 6) (right panel). Values are expressed as mean ± SEM; two-tailed Mann Whitney U test was performed for statistical analysis. (**C**) CD3 surface expression levels of naïve CD4+ T cells derived from 4 weeks SCD (white) or WD (gray) fed wild type mice following 24 h TCR stimulation with 0.5 μg/ml (low) and 1.5 μg/ml (high) soluble anti-CD3 antibody, assessed by anti-CD3 surface stainings utilizing peridinin chlorophyll-conjugated antibody. Pooled data from 2 independent experiments are shown. Repeated measures ANOVA with Bonferroni’s post-hoc test was performed for statistical analysis; *p < 0.05, **p < 0.01, ***p < 0.001.

Since FoxP3 expression and Treg cell commitment is determined by the strength of the TCR signaling in the thymus^29^, we hypothesized that diet-induced hypercholesterolemia may lead to increased Treg cell populations due to the altered interaction of T cells with APCs. Proliferation assays revealed that CD4+ CD25− T cells derived from WD fed mice were less responsive towards suppression with CD4+ CD25+ Treg cells from SCD fed animals (Supplemental Fig. 6A) and CD4+ CD25− T cell proliferation was promoted in the presence of irradiated APCs obtained from WD fed mice compared to those from SCD fed mice (Supplemental Fig. 6B). To test whether hypercholesterolemic conditions act stimulatory through enhanced T cell-APC interactions or inhibitory through the increased presence of functional tTreg cells, whole splenocyte proliferation assays of SCD and WD fed mice were performed. In essence, the proliferation of T cells obtained from WD fed mice was significantly higher than in those from SCD fed mice (Fig. 4A). Notably, expression of activation marker, such as ICOS, CD25, CD69 and CD44 in *ex vivo* isolated CD4+ and CD8+ T cells did not differ between the groups (data not shown), thus dietary cholesterol intake was unlikely to elicit full T cell activation. However, dietary cholesterol intake as well as LDLr deficiency significantly decreased the CD3 surface expression level of both, CD4+ CD25− and CD4+ CD25+ T cells (Fig. 4B). In line with this, CD4+ T cells derived from SCD fed mice required *in vitro* incubation with higher anti-CD3 concentrations to achieve a similar stimulation-dependent TCR internalization as in CD4+ T cells derived from WD fed mice (Fig. 4C). In summary, this suggests that hypercholesterolemia affects both, proliferation of T cells in the periphery and thymic development of Treg cells through altered TCR signaling.

### Dietary cholesterol intake increases TCR responsiveness

**Figure 5:**
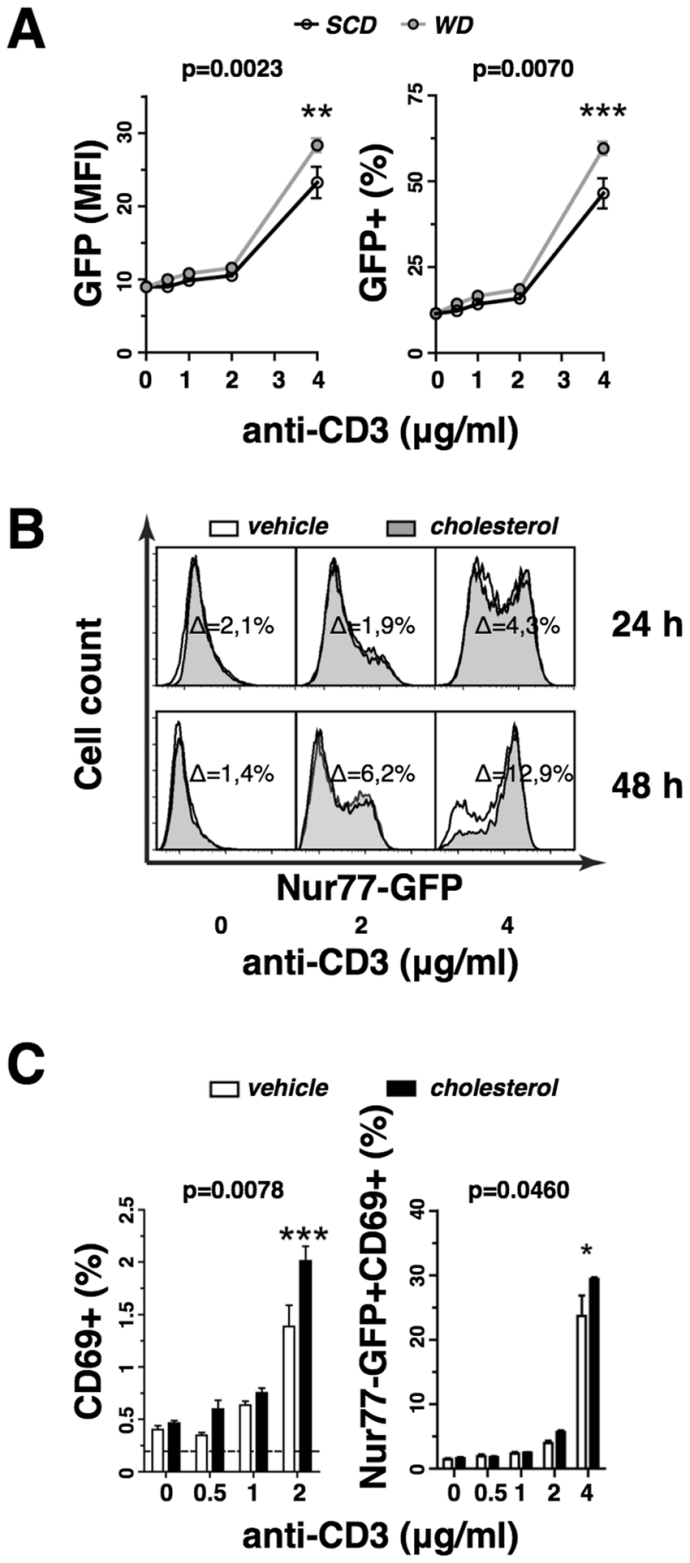
Cholesterol supplementation enhances T cell receptor signaling. (**A**) GFP expression level (left panel) and GFP+ population size (right panel) of isolated naïve CD4+ T cells derived from the spleens of 4 weeks cholesterol-free standard chow diet (SCD, white) or 0.15% cholesterol-containing Western diet (WD, gray) fed Nur77^GFP^ mice in response to 24 h polyclonal stimulation with soluble anti-CD3 antibody concentrations *in vitro* (n = 4). (**B**) GFP induction in isolated naïve CD4+ T cells derived from the spleens of Nur77^GFP^ mice following polyclonal stimulation with soluble anti-CD3 antibody concentrations in the presence of vehicle (white) or solubilized cholesterol (black shaded) for 24 and 48 h. The cholesterol-mediated gain of GFP+ T cell populations is shown. (**C**) Induction of CD69 expression in vehicle (white) or solubilized cholesterol (black) treated naïve CD4+ T cells isolated from the spleens of wild type mice following polyclonal stimulation with soluble anti-CD3 antibody concentrations (left panel) (n = 4) or Nur77^GFP^ mice following polyclonal stimulation with plate-bound anti-CD3 antibody concentrations (right panel) (n = 3). All values are expressed as mean ± SEM; two-way ANOVA with Bonferroni’s multiple comparison *post hoc* test was performed for statistical analysis; displayed p-values indicate the significant effect of the cholesterol supplementation on T cell activation marker expression. (The p-value for the effect of the antibody dilution is not shown); *p < 0.05, **p < 0.01, ***p < 0.001.

Next, we asked the question whether dietary-induced hypercholesterolemia would induce gene expression that has been linked to the TCR stimulation strength. For this, we analyzed the induction of Nur77 (Nr4a1), a transcription factor that can be used as surrogate marker for antigen receptor activation^30^, in response to anti-CD3 stimulation of naïve CD4+ T cells isolated from SCD or WD fed Nur77^GFP^ reporter mice. Dietary-induced hypercholesterolemia significantly enhanced TCR signaling and elicited both, increased Nur77-GFP+ T cell populations and increased Nur77-GFP expression levels per cell (Fig. 5A). This suggests that cholesterol-containing diet promotes the TCR responsiveness in CD4+ T cells.

### Cholesterol supplementation increases T cell activation *in vitro*

To pinpoint the effect of cholesterol on TCR signaling, we performed anti-CD3 stimulation assays with freshly isolated naïve T cells in the presence of exogenous cholesterol or vehicle control. Supplementation with solubilized cholesterol increased the total cellular cholesterol content moderately by 8.4% ± 4% without affecting the cell viability by more than 1%. However, cholesterol treatment enhanced Nur77-GFP expression in naïve Nur77^GFP^ T cells following stimulation with increasing soluble anti-CD3 antibody concentrations for 24 or 48 h in comparison to vehicle control (Fig. 5B). After 72 h *in vitro* cell culture, naïve T cells derived from mice fed cholesterol-containing diet or treated with cholesterol supplementation proliferated significantly stronger than controls with cholesterol-free treatments (Supplemental Fig. 7). Moreover, cholesterol supplementation *in vitro* increased significantly the expression of the activation marker CD69 in naïve wild type T cells stimulated with increasing soluble anti-CD3 antibody concentrations (Fig. 5C). Expression of CD69 coincided with Nur77-GFP induction and the Nur77-GFP+ CD69+ T cell population increased significantly upon cholesterol supplementation in naïve Nur77^GFP^ T cells following stimulation with increasing plate-bound anti-CD3 antibody concentrations. Thus, hypercholesterolemic conditions consistently enhanced the TCR stimulation strength and elicited increased expression of T cell activation markers, which increased the proliferative response.

## Discussion

Here we report that hypercholesterolemia promotes TCR stimulation in CD4+ T cells and facilitates proliferative T cell responses. These systemic changes are accompanied by enhanced Treg cell development in the thymus and increased FoxP3 expression in the periphery.

In this study, we analyzed CD4+ T cells that were either obtained from mice that were fed for at least four weeks with cholesterol-containing diet or isolated from splenocytes and incubated with cholesterol *in vitro*. In both experimental systems we observed that the cholesterol group had increased sensitivity towards TCR stimulation, evident by the lower anti-CD3 antibody concentrations needed to facilitate CD3 internalization, proliferation and induction of activation markers compared to the control group. However, the increased TCR signaling in WD fed mice did not result in increased expression of activation markers or differentiation of T cell subsets, except for Treg cells^28^. This suggests that cholesterol (i) promotes homeostatic TCR signaling to support Treg cells in the absence of antigen recognition and (ii) enhances TCR signaling to elicit T cell proliferation in the presence of antigen recognition.

*In vivo*, Treg cells depend on homeostatic TCR signaling to maintain their dynamic proliferative character^24^ and to express high levels of FoxP3^25^. This is in line with our findings that the Treg cell population increased relative to both, CD4+ T cells as well as total lymphocytes and elevated the FoxP3 expression level in WD fed mice compared to SCD fed littermates. Despite the enhanced FoxP3 expression and the increased suppressive capacity of the Treg cells in the hypercholesterolemic spleen, its T cell pool proliferated more vigorously in the presence of exogenous TCR stimulation, suggesting that WD treatment renders conventional T cells less susceptible for Treg cell-mediated suppression. Resistance towards Treg cell-mediated suppression is characteristically for pre-activated responder T cells^31^. Thus, T cells in hypercholesterolemic mice may experience subliminal pre-stimulation in the periphery as indicated by their increased ability to internalize CD3 and to induce Nur77-GFP expression. This may explain why in severely hypercholesterolemic *Ldlr*−/− mice (e.g. WD treatment for 24 weeks) T cells contribute to atherosclerosis development, although the splenic Treg cell compartment at this time point is greatly increased. Taken together, these results indicate that hypercholesterolemia enables T cells to react faster and/or stronger upon antigen recognition, which is in line with previous studies^6,32^.

In light of the multiple cholesterol-dependent functions in T cells and APCs, such as cell-intrinsic lipid metabolism^23,33^, plasma membrane organization^11^, TCR nanoclustering^11,34,35^ and endocytosis^18,20^, it is unlikely that dietary cholesterol would act unidirectionally *in vivo*. Therefore, our *in vivo* data may display the integral result of all hypercholesterolemia-mediated effects on T cells. However, our *in vitro* data demonstrated that isolated naïve CD4+ T cells were more efficiently stimulated in the presence of exogenous cholesterol after short time incubation with anti-CD3 antibody. Thus, circulating lipids may alter the plasma membrane cholesterol content and cholesterol may play a role in TCR signaling independent from its function as component for membrane biosynthesis and mediator of T cell-APC-interactions.

Manipulation of the lipoprotein metabolism affects immune homeostasis. Elevated high-density lipoprotein reduces T cell activation and proliferation and ameliorates the regulatory/effector T cell ratio in mice and men^36,37^. In contrast, elevated LDL increases T cell proliferation in *Ldlr* ^−/−^ mice fed cholesterol-containing diet^32^ and is associated with autoimmune diseases in humans^38^. Moreover, treatment with LDL, but not high-density lipoprotein, elicits activation-induced alternative splicing in human CD4+ T cells^39^. Thus, enhanced TCR signaling in hypercholesterolemia *in vivo* is probably mediated by LDL cholesterol.

Recently, we discovered that hypercholesterolemia promotes iTreg and Th17 cell differentiation by local TGF-β1 expression in the liver^28^. In contrast to the liver, T cell expression of Helios, Nrp-1 and Treg cell proliferation in the spleen did not change with increasing dietary cholesterol intake. Instead, we found that Western diet increased the population size and proliferation of FoxP3+ thymocytes, thus elevating the Treg cell population in secondary lymphoid organs. The thymic Treg cell selection depends on the TCR affinity towards autoantigens presented by APCs and thymic epithelial cells^40^. Thus, the accumulation of Treg cells in hypercholesterolemia may result from enhanced thymic selection due to enhanced TCR signaling. Interestingly, the thymic expression level of FoxP3 was not significantly increased, suggesting that the elevated FoxP3 expression level in splenic Treg cells from WD fed mice is later acquired through homeostatic TCR signaling in the periphery. Recent studies published by the Hedrick laboratory demonstrated that accumulation of cholesterol by genetic deficiency of ABCG1, the ATP-binding cassette transporter G1 promoting cellular cholesterol efflux, increased TCR signaling in the thymus and the periphery and elevated the population size of Treg cells^22,41^.

The inflammatory response in atherosclerosis is directed against the vascular deposition of lipoproteins and its derivatives. Thus, hypercholesterolemia provides (neo-)antigens that can trigger pro-atherosclerotic T cell responses^42^. In addition to elevated antigen concentrations, our results indicate that hypercholesterolemia changes antigen recognition through the TCR and promotes both, thymic selection and peripheral tolerance induction of Treg cells as well as the extent of effector T cell proliferation. Increasing the size and/or function of anti-inflammatory Treg cells as well as pro-inflammatory effector T cells destabilizes the immunological balance and increases the risk for impaired immune responses. Therefore, increased plasma cholesterol concentrations may disturb T cell homeostasis and predispose for aggravated T cell responses, as observed in numerous T cell-mediated comorbidities in CVD patients^43^. Further investigations are needed to elucidate how hypercholesterolemia affects specific T cell responses and how this is regulated through anti-inflammatory drugs in CVD treatment^44^.

In conclusion, we found that hypercholesterolemia affects the homeostatic as well as the proliferative TCR stimulation and increases the thymic as well as the peripheral FoxP3+ Treg cell population.

## Electronic supplementary material

Supplemental Figures
