# Supplementary material for: Hypercholesterolemia Enhances T Cell Receptor Signaling and Increases the Regulatory T Cell Population
Source: Sci Rep. 2017 Nov 15;7:15655. doi: 10.1038/s41598-017-15546-8 (PMC5688061; doi:10.1038/s41598-017-15546-8)
Supplement: Supplementary file 1 — Supplemental Figures [file 41598_2017_15546_MOESM1_ESM.pdf]

Supplemental Material

Hypercholesterolemia Enhances T Cell Receptor Signaling and Increases the Regulatory T Cell Population

Reiner K.W. Mailer[1], Anton Gisterå[1], Konstantinos A. Polyzos[1], Daniel F.J. Ketelhuth[1], Göran K. Hansson[1*]

[1]Cardiovascular Medicine Unit, Department of Medicine, Karolinska Institutet, Stockholm, Sweden

* **Correspondence:** Professor Göran Hansson: goran.hansson@ki.se

Supplemental Figure 1

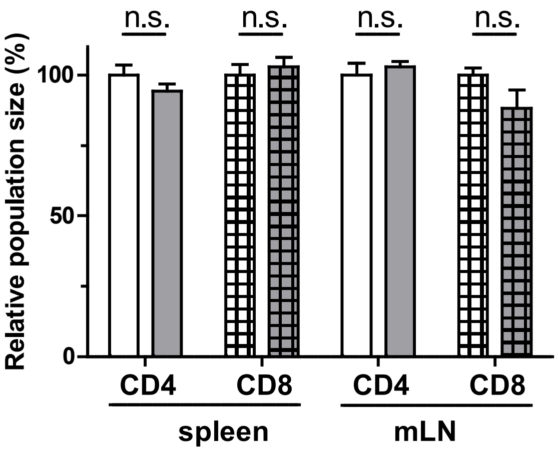

Peripheral lymphocyte populations are unaffected by hypercholesterolemia. CD4+ (plain) and CD8+ (cross-hatched) lymphocytes in spleen and mesenteric lymph nodes (mLN) from mice fed cholesterol-free standard chow diet (SCD, white) (n=4) or 0.15% cholesterol-containing Western diet (WD, gray) (n=5) for 4 weeks. Values are expressed as mean ± SEM; mean values obtained from SCD fed mice were set to 100%; two-tailed Mann Whitney U test was performed for statistical analysis.

Supplemental Figure 2

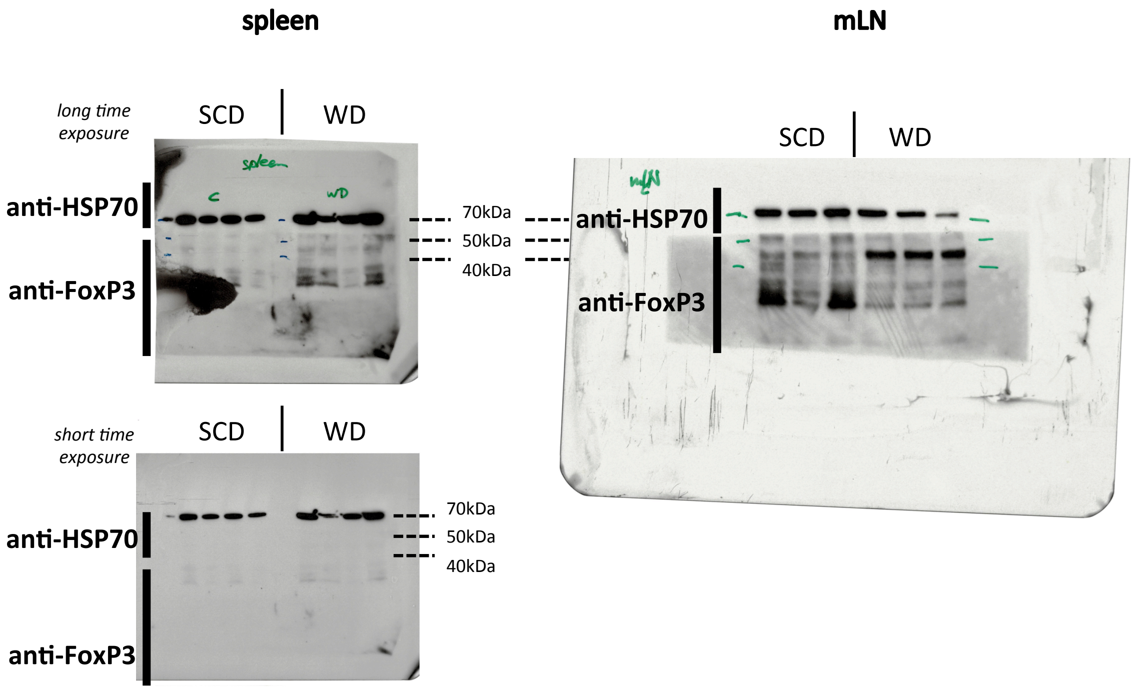

Dietary-induced hypercholesterolemia increases FoxP3 expression in spleen and mesenteric lymph nodes. Full-length immunoblots of splenocytes (left panel) and mesenteric lymph nodes (mLN) (right panel) from mice fed cholesterol-free standard chow diet (SCD) or 0.15% cholesterol-containing Western diet (WD) for 4 weeks as depicted in Figure 1D. Blotting membranes were cut and the upper part was incubated with anti-HSP70 (1B5, Assay designs) and the lower part was incubated with anti-FoxP3 (clone:eBio7979, eBioscience). Splenocytes band detection for both parts was performed with long (upper panel) and short (lower panel) exposition time.

Supplemental Figure 3

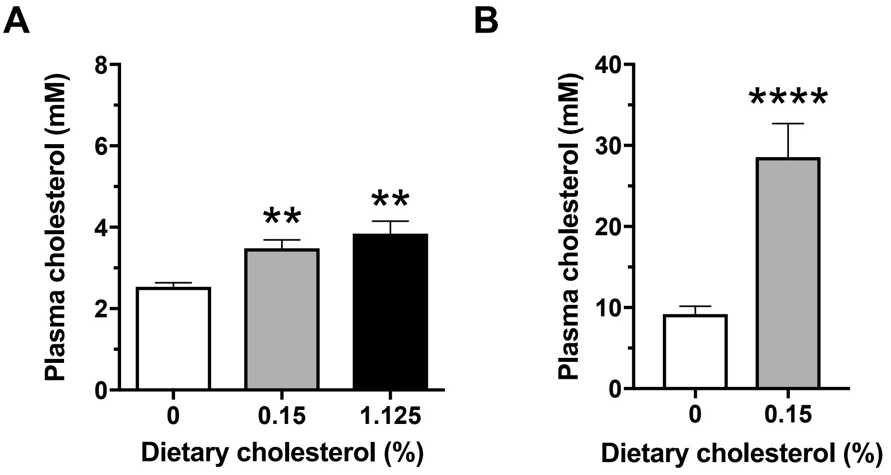

Hypercholesterolemia increases with dietary cholesterol content. Plasma cholesterol levels of (**A**) wild type mice fed 0% (white, n=11), 0.15% (gray, n=17) or 1.125% cholesterol-containing diet (black; n=7) and (**B**) *Ldlr*$^{-/-}$ mice fed 0% (white, n=4) or 0.15% (gray, n=13) cholesterol-containing diet for 4 weeks. Values are expressed as mean ± SEM; one-way ANOVA and Bonferroni's *post hoc* test and two-tailed Student's t-test was performed for statistical analysis; **$p<0.01$, ****$p<0.0001$.

Supplemental Figure 4

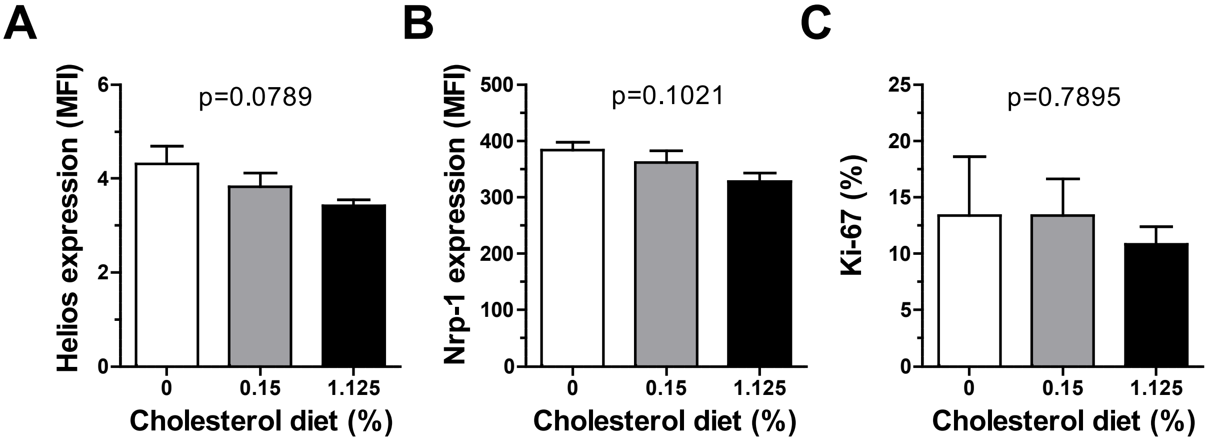

Hypercholesterolemia-induced Treg cell population increase is not related to induction or proliferation of peripherally induced Treg cells. Live FoxP3+CD4+ splenic Treg cells derived from mice fed 0% (white, n=3), 0.15% (gray, n=3) or 1.125% cholesterol-containing diet (black; n=6) for 4 weeks were analyzed for expression levels of (**A**) Helios, (**B**) Nrp-1 and for the percentage of proliferating cells (**C**). Values are expressed as mean ± SEM; Kruskal-Wallis ANOVA with Dunn's multiple comparison *post hoc* test was performed for statistical analysis.

Supplemental Figure 5

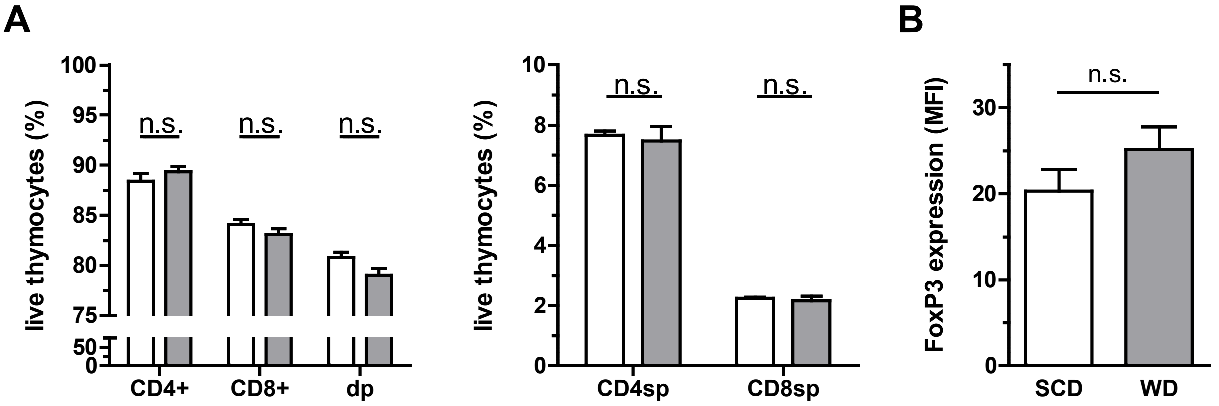

Dietary-induced hypercholesterolemia does not affect viability and size of thymic subpopulations. (**A**) Flow cytometry analysis of CD4+, CD8+, CD4+CD8+ double positive (dp), CD4+CD8- single positive (CD4sp) and CD8+CD4- single positive (CD8sp) subpopulations among live thymocytes in mice fed cholesterol-free standard chow diet (SCD, white) (n=3) or 0.15% cholesterol-containing Western diet (WD, gray) (n=4) for 4 weeks. (**B**) FoxP3 expression level of CD4+ thymocytes from mice fed SCD (n=6) or WD (n=7) for 4 weeks. Values are expressed as mean ± SEM; two-tailed Mann Whitney U test was performed for statistical analysis.

Supplemental Figure 6

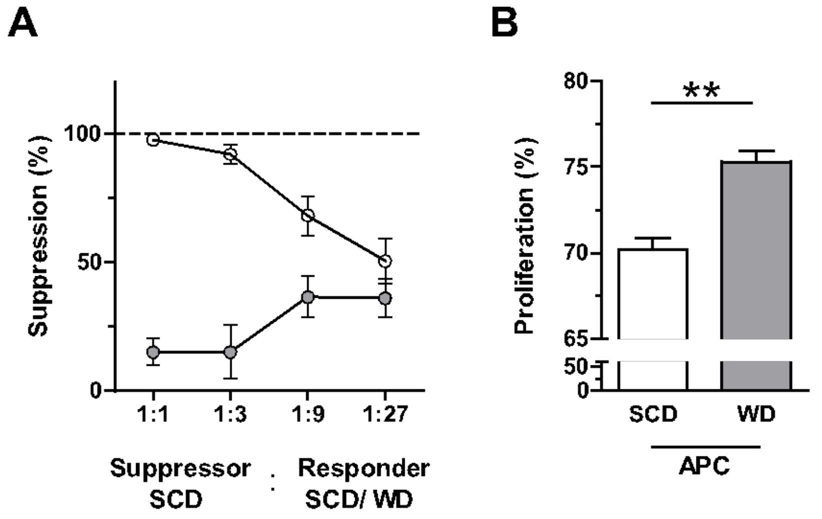

Resilience to suppression and proliferation is increased upon hypercholesterolemia. (**A**) Suppression of CFSE-labeled CD4+ responder T cells isolated from mice fed cholesterol-free standard chow diet (SCD, white) or 0.15% cholesterol-containing Western diet (WD, gray) for 4 weeks; CD4+CD25+ suppressor T cells and irradiated APCs are derived from SCD fed animals. Representative data from one out of two experiments with three technical replicates per condition are shown. Maximum and minimum suppression was set to 100% and 0%, respectively. Two-way ANOVA with Bonferroni's post-hoc test was performed; p-value indicates the significant effect of responder cell's diet (p-value for the effect of the suppressor cell dilution is not shown). (**B**) Proliferation of CFSE-labeled CD4+ responder T cells from SCD fed mice in the presence of irradiated APCs isolated from mice fed SCD (white) or 0.15% WD (gray) for 4 weeks. Percentages of dividing cell populations from one out of two experiments are shown (n=6); two-tailed Mann Whitney U test was performed for statistical analysis; **$p<0.01$.

Supplemental Figure 7

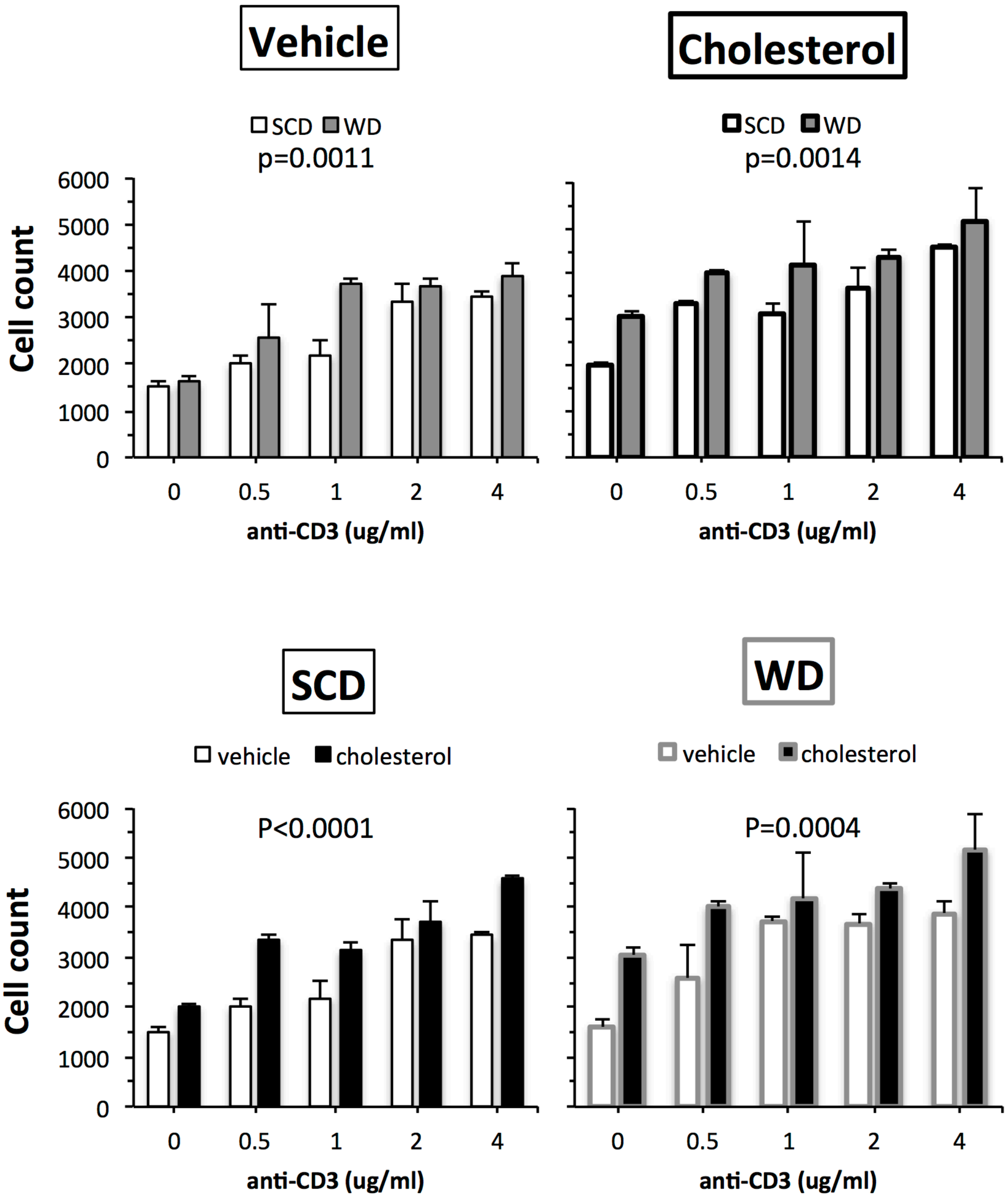

Dietary-induced hypercholesterolemia and cholesterol supplementation increase the proliferation of naïve T cells in vitro. FACS analysis of proliferating naïve T cells derived from 4 weeks cholesterol-free standard chow diet (SCD, white) or 0.15% cholesterol-containing Western diet (WD, gray) fed mice stimulated with 1 μg/ml anti-CD28 antibody and different soluble anti-CD3 concentrations in the presence or absence of 9 μg/ml supplemented cholesterol (black) for 3 days in vitro. Values are expressed as mean ± SD; two-way ANOVA with Bonferroni's multiple comparison *post hoc* test was performed for statistical analysis; displayed p-values indicate the significant effect of the diet (upper panel) on vehicle/cholesterol treated T cells and the significant effect of the cholesterol treatment (lower panel) on SCD/WD fed mice. (The p-value for the effect of the antibody dilution is not shown).

Supplemental Table 1: Experimental mouse models

Mouse strain	Genetic modification	Utilization
C57BL/6J	-	Wild type and control
Ldlr-/-	Targeted null/knockout (truncated and non-functional LDLr)	Hypercholesterolemia and experimental atherosclerosis model
$Nur77^{GFP}$	Nr4a1 transgenic (eGFP-hCre fusion protein)	Assessment of T cell receptor stimulation strength
DEREG	FoxP3 reporter (DTR-eGFP fusion protein on BAC)	Detection of FoxP3+ Treg cells

Supplemental Table 2: Experimental diet composition

Dietary content (%)	SCD	WD (0.15%)	WD (1.125%)
Cholesterol	0	0.15	1.125
Carbohydrates	60.1	43.9	45
Protein	14.5	17.2	23
Fat	4.5	21	20
Fibre	4.9	3.9	5.0
NaCl	0.4	0.3	0.1

Supplemental Table 3: Body weight, lymphoid organ weight and cellularity

Parameter	SCD	WD (0.15%)	*p*-value
Weight (g)	32.8 (32.3-33.2)	33.95 (31.3-39.3)	0.417
Thymus weight (mg)	48.9 (48.6-55.6)	54.2 (50.6-57.6)	0.328
Spleen weight (mg)	90.9 (84.5-91.4)	100.9 (80.6-153.5)	0.333
Thymocytes (x 10^6)	101 (96-120)	84 (80-107)	0.137
Splenocytes (x 10^6)	66 (54-82)	76 (66-93)	0.342

Values represent the median, range (min-max) and two-tailed Student’s t-test statistical analysis.
